# Development of a Large Set of Microsatellite Markers in Zapote Mamey (*Pouteria sapota* (Jacq.) H.E. Moore & Stearn) and Their Potential Use in the Study of the Species

**DOI:** 10.3390/molecules200611400

**Published:** 2015-06-22

**Authors:** Renée S. Arias, Jaime Martínez-Castillo, Victor S. Sobolev, Nasib H. Blancarte-Jasso, Sheron A. Simpson, Linda L. Ballard, Mary V. Duke, Xiaofen F. Liu, Brian M. Irish, Brian E. Scheffler

**Affiliations:** 1 USDA-ARS National Peanut Research Laboratory, 1011 Forrester Dr. S.E., Dawson, GA 39842, USA; E-Mails: renee.arias@ars.usda.gov (R.S.A); victor.sobolev@ars.usda.gov (V.S.S.); 2 Centro de Investigación Científica de Yucatán A.C., Unidad de Recursos Naturales, Calle 43 No. 130, Colonia Chuburná de Hidalgo CP 97200, Mérida, Mexico; E-Mail: hiram.blancarte@cicy.mx; 3 USDA-ARS Genomics and Bioinformatics Research Unit, 141 Experiment Station Rd., Stoneville, MI 387761, USA; E-Mails: sheron.simpson@ars.usda.gov (S.A.S.); linda.ballard@ars.usda.gov (L.L.B.); mary.duke@ars.usda.gov (M.V.D.); fanny.liu@ars.usda.gov (X.F.L.); brian.scheffler@ars.usda.gov (B.E.S.); 4 USDA-ARS Tropical Agriculture Research Station, 2200 Pedro Albizu Campos Ave, Mayaguez, PR 00680, USA; E-Mail: brian.irish@ars.usda.gov

**Keywords:** blast analysis, founder effect, genetic diversity, germplasm, domestication, Mexico, SSR markers, genetic structure

## Abstract

*Pouteria sapota* is known for its edible fruits that contain unique carotenoids, as well as for its fungitoxic, anti-inflammatory and anti-oxidant activity. However, its genetics is mostly unknown, including aspects about its genetic diversity and domestication process. We did high-throughput sequencing of microsatellite-enriched libraries of *P. sapota*, generated 5223 contig DNA sequences, 1.8 Mbp, developed 368 microsatellites markers and tested them on 29 individuals from 10 populations (seven wild, three cultivated) from Mexico, its putative domestication center. Gene ontology BLAST analysis of the DNA sequences containing microsatellites showed potential association to physiological functions. Genetic diversity was slightly higher in cultivated than in the wild gene pool (*H*_E_ = 0.41 and *H*_E_ = 0.35, respectively), although modified Garza–Williamson Index and Bottleneck software showed evidence for a reduction in genetic diversity for the cultivated one. Neighbor Joining, 3D Principal Coordinates Analysis and assignment tests grouped most individuals according to their geographic origin but no clear separation was observed between wild or cultivated gene pools due to, perhaps, the existence of several admixed populations. The developed microsatellites have a great potential in genetic population and domestication studies of *P. sapota* but additional sampling will be necessary to better understand how the domestication process has impacted the genetic diversity of this fruit crop.

## 1. Introduction

The genus *Pouteria* Aublet (*Sapotaceae*) has 325 species, many of them known for their edible fruit and quality wood. It is a non-monophyletic genus composed of at least three distinct evolutionary lineages [1,2]. One important species of this genus is *P. sapota* ((Jacq.) H.E. Moore & Stearn), commonly known as zapote mamey, mamey or mamey rojo [3]. The natural habitat of *P. sapota* is the tropical and sub-tropical evergreen forests distributed on Mexico’s Atlantic slope from North Veracruz to Tabasco state as well as in Mexico’s Pacific slope from Jalisco to Chiapas, continuing through Central America up to Panama, and from 0–1300 m above sea level [4,5]. Multiple wild and cultivated populations of *P. sapota* have been recognized, mainly by fruit characteristics because wild fruits are significantly smaller than cultivated ones. However, there are no formal studies about the existence of subspecies or botanical varieties. The lowlands from Mexico and Central America are the putative center of domestication of *P. sapota* [5,6]. Mexico is the most important producer of zapote mamey in the world, being Yucatan State the main producing area [7]. In this country, the traditional method of production is still in small orchards or by harvesting wild trees in the regions where they exist, whereas grafting is generally employed in small plantations or traditional home gardens [6]. At present, zapote mamey has been introduced as a fruit crop in South America, the Caribbean, Europe, Asia, Australia, and among other regions on the world [6].

Currently, there is abundant information about the chemical composition of zapote mamey for its high nutritional value as the red fruits contain unique ketocarotenoids, such as cryptocapsinepoxide [8] and sapotexanthin [9], all precursors of vitamin A. The pulp of the fruit can have up to 247 mg of ascorbic acid per 100 g dry weight [10], and the main fatty acid in seed (oleic acid) can be up to 60% of the seed oil [11]. Traditionally, oils extracted from the seeds of *P. sapota* have been used for multiple medicinal purposes, including the effective control of the fungal skin dermatitis [12]. In fact, antimicrobial, trypanocidal, insecticidal, anti-inflammatory and anti-oxidant activities have been observed in compounds extracted from fruits, leaves, and bark of many other *Pouteria* species [13]. In contrast, little is known about how the domestication process of *P. sapota* and to what extend this may have impacted its genetic diversity in populations of vegetatively propagated fruit trees [14,15]. In fact, there is a lack of codominant molecular markers for this species that could help answer these questions. Therefore, genetic diversity has been mainly studied using morphological and biochemical characters of fruits from cultivated germplasms [16,17,18,19]. In recent years, studies have incorporated the use of dominant molecular markers [Random Amplified Polymorphic DNA (RAPD) and Amplified Fragment Length Polymorphism (AFLP)], but they only have analyzed genetic relationships among cultivated germplasms [20,21,22]. Thus, no genetic variations have been associated to the geographic origin of the samples, and no estimators of genetic diversity have been reported because dominant markers such as RAPDs and AFLPs cannot properly estimate genetic diversity.

Currently, there is a lack of molecular tools to study *Pouteria* species, and data mining is not an option as there is only one entry of 443 bp (GQ402492.1) for *P. sapota* in NCBI, GenBank. In absence of sequenced genomes, microsatellite-enrichment of genomic libraries has been shown effective to develop molecular markers for genetic studies [23,24]. Microsatellites, also known as simple sequence repeats (SSRs), are a widely applied class of codominant molecular markers used in molecular breeding, genetic conservation, and population genetics [25,26]. Therefore, the objectives of the present study were: (1) develop a large set of SSR molecular markers specific for *P. sapota*; and (2) to analyze, for the first time, the structure and genetic diversity of wild and cultivated populations of *P. sapota* collected in Southeast of Mexico, the putative domestication center of this species.

## 2. Results and Discussion

### 2.1. Development of Microsatellite Markers

Sequencing of *P. sapota* microsatellite-enriched libraries of genomic DNA resulted in 5223 assembled contigs with an average length of 345 bp (min: 94 bp; max: 1173 bp), and an average reads per contig of 4.4 (min 2; max 127). Until now, there was no information about the genetics of *P. sapota*. In the sequenced libraries we found 1452 contigs with significant hits in BLAST analysis; 28% of them with similarity to *Vitis vinifera*, 8% to *Populus trichocarpa*, 5.5% to *Glycine max*, and the rest had similarity to other tropical and non-tropical plant species. A total of 685 microsatellites were detected in 379 contigs using the software SSR Finder, 384 primer sets were designed and tested, 96% (368) of these primer sets resulted in effective amplification, and 205 SSR were polymorphic and amplified all 31 samples. Further analysis of SSR-containing contigs of polymorphic loci showed that 55 of the 368 developed SSR markers had significant hits (Expected values ≤ 1 × 10^−4^) on BLAST to Gene Ontology (BLAST2GO) analysis (Supplementary Materials). These included those potentially related to hormones and organ development (stv-pos_2881_a; stv-pos_0334_a; stv-pos_1840_b; stv-pos_1465_a; stv-pos_2304_a), biotic/abiotic stress response (stv-pos_0551_a; stv-pos_2461_a; stv-pos_2375_a; stv-pos_2408_a; stv-pos_0633_a; stv-pos_1332_a and stv-pos_2565_a), chloroplast formation and phototropism (stv-pos_1364_a; stv-pos_2002_a; stv-pos_1602_a; stv-pos_2590_a and stv-pos_3313_a), phosphorous uptake by roots (stv-pos_2091_a), folate synthesis (stv-pos_2582_a), seed maturation (stv-pos_1539_a), flavonoid synthesis or fruit color (stv-pos_1628_a andtv-pos_1632_a), and phytoalexin synthesis (stv-pos_0983_a).

**Figure 1:**
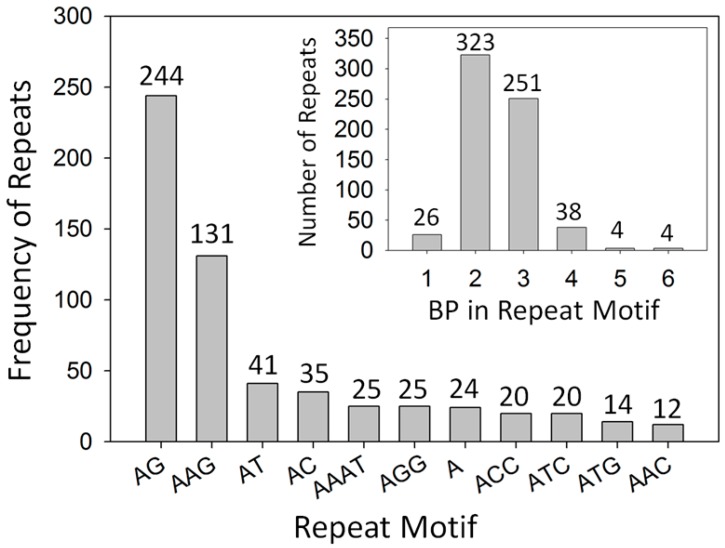
Motifs and frequency of repeats detected in *Pouteria sapota* microsatellite-enriched libraries. Reverse complementary sequences were combined as one motif.

Individual contigs often presented more than one microsatellite, and these simple-sequence repeats were separated by one or more basepairs (bp). Apparently, when the distance in bp between two microsatellites within a contig was ≤10 bp (normally called imperfect repeats), 64%–90% of the markers within that contig were polymorphic. When the distance between microsatellites within a contig was longer, for example 11–343 bp then, ≤50% of the markers derived from those contigs were polymorphic (Supplementary Materials). Only 11 of the 368 microsatellites (2.9%) apparently deviated from Hardy–Weinberg equilibrium. Most repeat motifs detected were 2 bp long, and the most frequent motifs on the sequenced DNA were AG and AGG (Figure 1). To simplify the recording of the repeat motifs, those that were circular permutations and reverse complements of each other were grouped together as one type, *i.e.*, AAC, ACA, CAA, GTT, TGT and TTG were recorded as AAC. The total length of individual microsatellite sequences was between 8 and 32 bp. About half (183/379) of the contigs containing microsatellites had more than one motif (between 2 and 6), in those cases the distances between repeats within each contig were between 0 and 343 bp. No significant correlation (*r* = −0.449, non significant) was observed between the total repeat length (8–32 bp) and the polymorphism of the markers using 31 DNA samples for the 193 loci that had a single microsatellite per contig. The discriminating power of the markers was calculated with unique pattern informative combinations (UPIC) software [27] and the results are provided (Supplementary Materials). The smallest combination of markers that could discriminate all 31 samples was calculated using UPIC, and the 10 markers in that combination were: stv-pos_01505_a, stv-pos_00588_a, stv-pos_01512_a, stv-pos_00286_a, stv-pos_02644_b, stv-pos_00756_a, stv-pos_02013_a, stv-pos_01677_a, stv-pos_01580_a, and stv-pos_01632_a, with a total sum of UPIC scores equal to 44 (Supplementary Materials).

### 2.2. Genetic Diversity and Founder Effect

All estimators of genetic diversity were slightly higher in the cultivated than in the wild gene pool of *P. sapota* from Mexico (Table 1). This is an unexpected result considering the smaller number of cultivated individuals analyzed (Table 1). At present, there are no studies reporting genetic diversity in wild and cultivated populations of *P. sapota* in the world. Genetic diversity levels have been reported for others species within *Sapotaceae*. For example, high levels of genetic diversity were reported in 294 individuals (*H*_E_ = 0.86) of a wild population of masaranduba (*Manilkara huberi* (Ducke) Standl) from Amazonas using SSRs [28]. Heterozygosity, *H*_E_ = 0.951, of 20 wild individuals of sapodilla (*M. sapota* (L.) P. Royen) from Mexico was estimated using SSRs [29]. Heterozygosity, *H*_E_ = 0.59, for three morphotypes of argan (*Argania espinosa* (L.) Skeels) from Morocco was determined using SSRs [30]. Furthermore, heterozygosity, *H*_E_ = 0.35, for seven populations of Puerto Rican bully (*Sideroxylon portoricense* subsp. *Minutiflorum* Pittier) from Mexico was obtained using Inter Simple Sequence Repeats (ISSRs) [31]. Though compared with those studies, relatively low levels of genetic diversity were observed in the present work on *P. sapota* from Mexico (*H*_E_ = 0.34). However, we believed this estimation of the genetic diversity in these populations is accurate given the large number of loci (approx. 200) analyzed here, and the accuracy of allele calling (1 bp differences detected) when using capillary electrophoresis.

**Table 1:** Estimators of genetic diversity and bottleneck of wild and cultivated gene pools of *Pouteria sapota* from Mexico, using 205 microsatellite loci.

Gene Pools	N	A	*H*_O_ ± SD	*H*_E_ ± SD	G–W ± SD
Wild	20	2.98	0.32 ± 0.25	0.35 ± 0.20	0.49 ± 0.31
Cultivated	9	3.00	0.39 ± 0.24	0.41 ± 0.17	0.42 ± 0.32
All Mexico	29	2.99	0.31 ± 0.24	0.34 ± 0.21	0.46 ± 0.31

N, Sample size; A, Average number of alleles per locus using rarefaction methods; *H*_O_, Observed heterozygosity; *H*_E_, Expected heterozygosity; G–W, Garza–Williamson Index; SD, Standard deviation.

The higher level of genetic diversity observed in the cultivated gene pool of *P. sapota* could suggest that there was no founder effect during the domestication process of this species. However, when the founder effect was evaluated using the modified Garza–Williamson index, it was higher in the wild (0.49) than in the cultivated (0.42) gene pool (Table 1), indicating the possible existence of a founder effect in this last gene pool. This result was supported when founder effect was evaluated using Bottleneck software (cultivated gene pool, *P*-value = 0.00003). Another finding supporting a founder effect is that in 62 SSR loci analyzed, one or more alleles observed in the wild individuals, mainly in JC population, were not detected in cultivated individuals; this included markers stv-pos_1117, stv-pos_1602, stv-pos_1677, stv-pos_2282, stv-pos_2375, stv-pos_2461, stv-pos_2565, stv-pos_2755, stv-pos_2977, stv-pos_3228, stv-pos_3569, and stv-pos_3748 (Supplementary Materials). The existence of a founder effect in domesticated plants is something common in seed crops [32]. However, zapote mamey is a fruit crop propagated vegetatively by the farmers from Mexico [6]. The domestication process in vegetatively propagated cultivated trees likely involved few recombination and sexual cycles, resulting in cultivated populations that may not have diverged significantly from their wild progenitor [33].

A more alarming and also possible explanation for the lower level of genetic diversity observed in the wild gene pool is that this one had suffered a genetic erosion process because of human activities. The region where the wild populations of *P. sapota* were collected is a hot spot for potential species extinction as a result of urbanization, agriculture and other land uses [34]. Thus, important germplasms could vanish before their potential uses or benefits are described. Since the goal of gene banks is to preserve agricultural biodiversity, attention should be paid to the 62 SSR markers that showed alleles in wild but not in cultivated populations. BLAST analysis for some of these 62 markers showed potential homology to interesting biological functions, indicating that these markers can serve as a tool to identify valuable germplasms.

### 2.3. Genetic Structure and Cluster Analysis

There was a low genetic differentiation among the 10 studied populations of *P. sapota* from Mexico. We found an *F*_ST_ = 0.136 among all populations, ranging between pairs of populations from 0.01 to 0.18 (Table 2). Analysis of Molecular Variance (AMOVA) confirmed these results; only 3.8% of the total variation was between wild and cultivated gene pools (Table 3). As has been shown, the domestication process in vegetatively propagated cultivated trees can result in cultivated populations that do not significantly diverge from their wild progenitor [34]. Also, this low differentiation could be a result of genetic flow. We found high levels of genetic flow among populations, ranging between pairs of populations from 1.75 to 74.1 (Table 2). Though, there are no studies reporting genetic structure in *P. sapota*, a few reports about the genetic differentiation in other *Sapotaceae* species [31] exist; but they are not comparable with the present study as they did not analyze wild and cultivated populations, as shown here.

**Table 2:** Values of *F*_ST_ (above diagonal) and genetic flow (below diagonal) between pairs of populations of *Pouteria sapota* from Mexico, using 205 SSR loci.

Populations	AA	JC	NP	PJ	UR	WH	YJ	AT	DZ	OX
AA	***	0.11	0.03	0.03	0.11	0.03	0.06	0.04	0.05	0.11
JC	3.89	***	0.11	0.16	0.18	0.15	0.12	0.13	0.14	0.22
NP	14.63	3.75	***	0.01	0.10	0.04	0.02	0.06	0.08	0.14
PJ	13.70	2.61	74.10	***	0.07	0.02	0.07	0.03	0.05	0.12
UR	3.93	2.25	4.40	5.90	***	0.12	0.02	0.10	0.14	0.21
WH	12.20	2.79	9.71	17.98	3.61	***	0.07	0.07	0.12	0.16
YJ	6.72	3.42	21.95	5.97	23.38	6.57	***	0.08	0.13	0.18
AT	12.00	3.21	7.79	15.18	4.10	6.16	5.44	***	0.08	0.06
DZ	8.42	2.92	5.68	8.11	2.98	3.52	3.16	5.64	***	0.06
OX	3.95	1.75	2.99	3.55	1.87	2.53	2.14	7.06	6.92	***

***: Diagonal line dividing the *F*_ST_ and M values; The names of the populations and geographic origin are as listed in Table 4.

**Table 3:** AMOVA of 10 wild and cultivated populations of *Pouteria sapota* from Mexico, using 205 microsatellite loci.

Source of Variation	Degree of Freedom	Sum of Squares	Variance Components	Percentage of Variation
Among groups (wild and domesticated gene pools)	1	59.848	0.99087 Va	3.82
Among populations within groups	8	279.256	2.08706 Vb	8.05
Within populations	48	1097.000	22.85417 Vc	88.13
Total	57	1436.103	25.93210	

Fixation Indices: FSC = 0.08368; FST = 0.11869; FCT = 0.03821.

**Figure 2:**
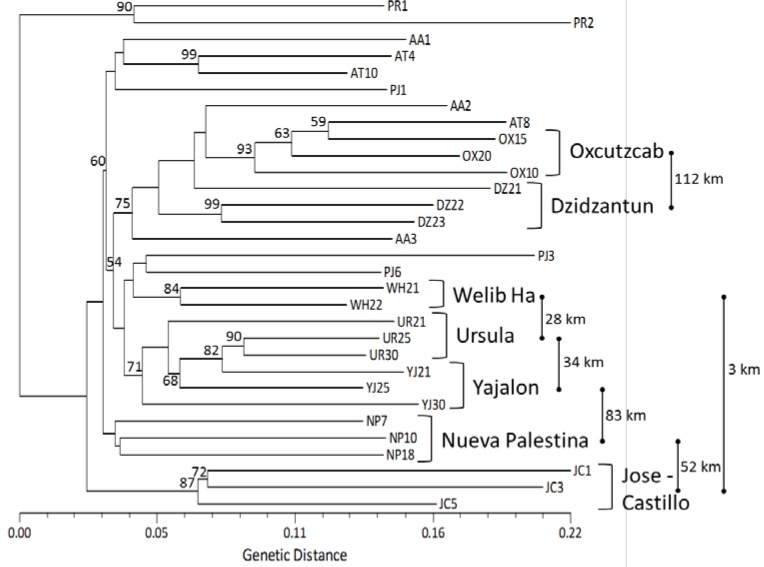
Neighbor-Joining analysis of seven wild and three cultivated populations of *Pouteria sapota* from Mexico and two individuals from Puerto Rico, using 205 polymorphic SSR loci. Bootstrap confidence values higher than 50% (50,000 resampling) are shown at the nodes. Symbols used for each of the populations are described in Table 4. 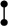: distance between two locations in kilometers, not at scale.

Neighbor-Joining analysis showed a general clustering pattern according to the geographical origin of the populations. Eight of the 11 populations were clearly discriminated with high bootstrap values (Figure 2). The two individuals from Puerto Rico were genetically distant from the rest of the individuals from Mexico. Although no clear separation was observed between wild or cultivated individuals, in general, most individuals grouped with others from the same geographic area, *i.e.*, wild (WH, UR, YJ, NP and JC) or cultivated (PR, OX and DZ). Individuals from populations AA and PJ (both wild), and AT (cultivated) were not clearly grouped by their geographic origin.

**Figure 3:**
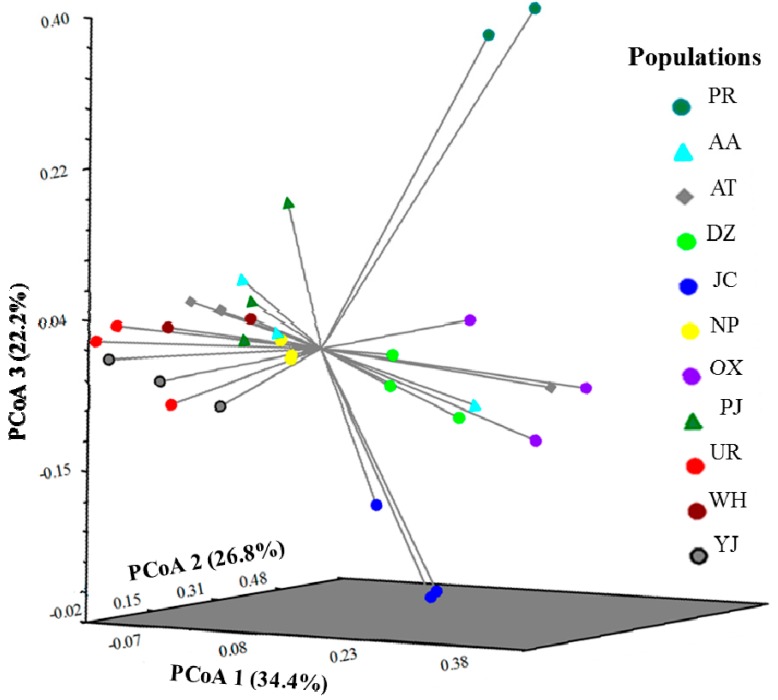
Three dimensional principal coordinate analysis (3D PCoA) of 10 populations of *Pouteria sapota* collected in Mexico and two individuals from Puerto Rico, using 205 microsatellite loci. The names of the populations and geographic origin are as listed in Table 4.

The clustering pattern showed by the N-J analysis seems to be consistent with that obtained with the three-dimensional principal coordinate analysis (3D PCoA) (Figure 3). The two individuals from Puerto Rico are clearly separated from individuals of Mexico. Individuals from nine out of eleven populations grouped with other individuals from the same population, except for AA (wild) and AT (cultivated). In this analysis, the first three dimensions (dim-1, dim-2 and dim-3) explained 32.4%, 26.8% and 22.2% of the genetic variation, respectively (Figure 3).

**Figure 4:**
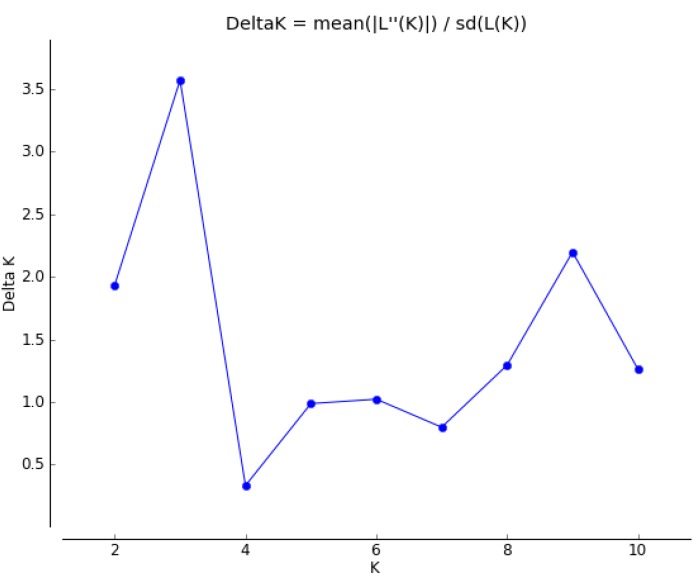
Graph of delta K values to determine the ideal number of groups present in 10 populations of *Pouteria sapota* from Mexico, using 205 microsatellite loci and the Evanno method implemented in the STRUCTURE HARVESTER program.

**Figure 5:**
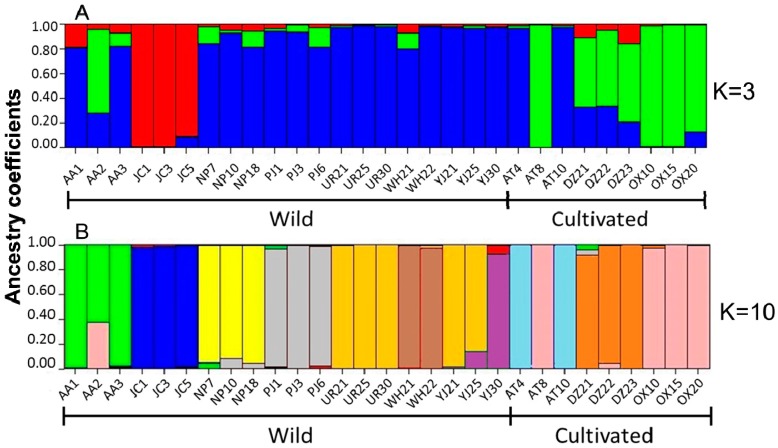
Proportion of estimated ancestry of seven wild and three cultivated populations of *Pouteria sapota* from Mexico, using the program STRUCTURE and 205 microsatellite loci. (**A**) K = 3; (**B**) K = 10. The names of the populations and geographic origin are as listed in Table 4.

The clustering pattern of the Mexican *P. sapota* populations was further assessed on the basis of the assignment tests carried out with STRUCTURE. The Evanno method indicated an ideal K = 3 (Figure 4). Figure 5A shows the clustering pattern of wild and cultivated populations when K = 3. As in the N-J and 3D PCoA analyses, no clear separation was observed between wild and cultivated individuals. In this graph, we can see that the Jose Castillo wild population was different from the other wild ones. This finding could explain the ideal K = 3 found with the Evanno method, instead of K = 2, considering only the existence of wild and cultivated gene pools. Also, Figure 5A show that one individual of AA wild population (AA2) shared ancestry with DZ and OX cultivated populations, whereas two individuals from AT cultivated population (AT4, AT10) shared ancestry with several wild populations.

**Table 4:** *Pouteria sapota* accessions used in this study.

ID	Origin	Accession	Location	Coordinates
PR1	cultivated	Lorito #2; TARS: 17854	B-IV, T-2 F-1, Isabela, Puerto Rico	18°30′47″ N 67°04′12″ W
PR2	Cultivated	Pace; TARS: 17866	B-IV, T-2, F-13, Isabela, Puerto Rico	
AA1	wild	(J)_1	Agua Azul, Mexico	17°15.494′ N 92°06.824′ W
AA2	wild	(J)_2	Agua Azul, Mexico	
AA3	wild	(J)_3	Agua Azul, Mexico	
JC1	wild	(E)_1	Jose Castillo, Mexico	17°23.807′ N 91°48.748′ W
JC3	wild	(E)_3	Jose Castillo, Mexico	
JC5	wild	(E)_5	Jose Castillo, Mexico	
NP7	wild	(L)_7	Nueva Palestina, Mexico	16°57.810′ N 91°37.665′ W
NP10	wild	(L)_10	Nueva Palestina, Mexico	
NP18	wild	(L)_18	Nueva Palestina, Mexico	
PJ1	wild	(K)_1	Penjamo, Mexico	17°26.041′ N 91°37.665′ W
PJ3	wild	(K)_3	Penjamo, Mexico	
PJ6	wild	(K)_6	Penjamo, Mexico	
UR21	wild	(B)_21	Ursula, Mexico	17°18.416′ N 92°02.997′ W
UR25	wild	(B)_25	Ursula, Mexico	
UR30	wild	(B)_30	Ursula, Mexico	
WH21	wild	(C)_21	Welib Ha, Mexico	17°22.521′ N 91°47.893′ W
WH22	wild	(C)_22	Welib Ha, Mexico	
YJ21	wild	(A)_21	Yajalon, Mexico	17°13.475′ N 92°21.184′ W
YJ25	wild	(A)_25	Yajalon, Mexico	
YJ30	wild	(A)_30	Yajalon, Mexico	
AT4	Cultivated	(G)_4	Atasta, Mexico	18°36′58.9″ N 92°9′12.13″ W
AT8	Cultivated	(G)_8	Atasta, Mexico	
AT10	Cultivated	(G)_10	Atasta, Mexico	
DZ21	Cultivated	(M)_21	Dzidzantun, Mexico	21°14′53.5″ N 89°1′59.65″ W
DZ22	Cultivated	(M)_22	Dzidzantun, Mexico	
DZ23	Cultivated	(M)_23	Dzidzantun, Mexico	
OX10	Cultivated	(F)_10	Oxcutzcab, Mexico	20°18′10″ N 89°25′0″ W
OX15	Cultivated	(F)_15	Oxcutzcab, Mexico	
OX20	Cultivated	(F)_20	Oxcutzcab, Mexico	

Figure 5B shows the clustering pattern of wild and cultivated Mexican populations when K = 10 (considering the 10 sites collected in Mexico as different populations). In this graph, individuals from nine out of 10 Mexican populations were assigned according to their geographic origin. Also, we can see individuals admixed or migrants: One individual from AA wild population (AA2) had significant contribution of genetic material from OX cultivated population; one individual from AT cultivated population (AT10) was identical to those of population OX; and two individuals from YJ wild population (YJ21, YJ25) showed high genetic contribution from population UR.

Combining the results of the three cluster analyses performed (N-J, 3D PCoA, and STRUCTURE), it seems that the populations of *P. sapota* from Mexico are grouped by their geographical origin. However, Mantel test did not show the existence of a geographical isolation among the studied populations (all populations, *r* = 0.369, *p*-value = 0.09). The existence of admixed population shown by the STRUCTURE analyses is probably responsible for this result. These admixed populations hindered their grouping in the N-J and 3D PCoA analyses (Figure 2 and Figure 3). In zapote mamey, most of molecular studies have been conducted to analyze the genetic relationships in cultivated germplasms. Using AFLP on 41 accessions from Cuba and Yucatan, no association to geographic area was found [20]. RAPDs and RAMP analyses on 14 accessions (13 from Guerrero and one from Yucatan, Mexico), did not distinguish the geographic origin of the accessions [21]. RAPDs on 15 trees in the state of Morelos, Mexico, showed that one of those trees was genetically dissimilar from the rest, and probably from a different geographic origin [22]. Hence, the microsatellite markers reported in the study allowed, for the first time, the clear discrimination by geographic origin of wild populations of *P. sapota* that were as close as 3 km apart (WH and JC) and many others that were between 28 to 112 km apart (Figure 2, Table 4).

## 3. Experimental Section

### 3.1. Plant Material, DNA Extraction, and Isolation of Microsatellites

Analysis included 29 individuals of *P. sapota* collected in Mexico. Of these, 20 individuals were collected directly in the field from seven wild populations and nine individuals were collected in traditional home gardens from three Mayan towns of the Yucatan peninsula (Table 4). The classification of these 29 individuals in wild and cultivated forms was based, essentially, on the site of collection (wild *vs*. traditional home gardens) and fruit size. Also, we included two individuals from the collection at USDA-ARS Tropical Agriculture Research Station (TARS) in Puerto Rico. DNA was extracted from lyophilized young leaves using DNeasy Plant Maxi kit (QIAGEN, Valencia, CA, USA). The accessions of *P. sapota* from TARS collection were used to generate separate SSR-enriched libraries following the protocol of Techen [24], briefly described here. DNA was digested with restriction enzymes AluI, HaeIII, DraI, RsaI, and HpyCH4IV (New England Biolabs, Ipswich, MA, USA). The DNA fragments were A-tailed with Taq-DNA Polymerase (Promega, Madison, WI, USA) in the presence of dATP for 2 h and then ligated for 3 h at 16 °C to a linker made from oligonucleotides (oligos) SSRLIBF3, 5′-CGGGAGAGCAAGGAAGGAGT-3′ and SSRLIBR3, 5′-Phos-CTCCTTCCTTGCTCTCTCCCGAAAA-3′ [24]. The ligated fragments were purified with MinElute (QIAGEN) and amplified by 20 cycles of PCR by using primer SSRLIBF3 and High Fidelity DNA Polymerase (Invitrogen, Carlsbad, CA, USA) at 94 °C for 30 s, 60 °C for 30 s, and 68 °C for 90 s. The amplified products were hybridized to three groups of biotinylated oligo repeats [23]. Sequences containing repeats were captured using streptavidin-coated magnetic beads M-270 (Invitrogen) [35]. After binding, the beads were washed with 0.5 × standard saline citrate (SSC) for 5 min. Elution of the DNA from the biotinylated oligos was done with 100 μL of MilliQ water (Millipore, Billerica, MA, USA) at 96 °C for 5 min. The eluate was PCR amplified for 10 cycles, as indicated for the ligation step. PCR products were sequenced by pyrosequencing in 1/8th of a plate 454-GS FLX (Roche Diagnostics, Indianapolis, IN, USA) by using GS Titanium Sequencing kit XLR70 (70 by 75 Titanium Pico-Titer Plates, Roche, Branford, CT, USA; 200 cycles). Sequences were assembled with 454 gsAssemby version 2.0 (Roche). Repeats were searched using SSRFinder [36], and primers were designed using Primer3 [37] with stringent parameter conditions: Annealing temperature 63 °C ± 1 °C, length 24-bp, 3′ GC clamp, and 5 bp maximum overlap of repeat within the primer. Assembled contigs resulting from high-throughput sequencing were screened using BLAST (Basic Local Alignment Search Tool) [38], BLASTn and MegaBLAST. Analysis of gene ontology was performed for the 379 repeat-containing DNA sequences using BLAST2GO [39]. 

### 3.2. Fingerprinting

A total of 384 primer sets were designed and used to screen 31 DNA samples of *P. sapota* listed in Table 2. Forward SSR primers were 5′ tailed with the sequence 5′-CAGTTTTCCCAGTCACGAC-3′ to permit product labeling [40], and reverse primers were tailed at the 5′ end with the sequence 5′-GTTT-3′ [41]. Primer 5′-CAGTTTTCCCAGTCACGAC-3′ labeled with 6-carboxy-fluorescein (FAM) (Integrated DNA Technologies, Inc., Coralville, IA, USA) was used for amplification of 15-ng DNA by using Titanium TaqDNA Polymerase (Clontech, Mountain View, CA, USA) in 5 μL reactions on an M&J thermal cycler (Bio-Rad Laboratories, Hercules, CA, USA) at 95 °C for 1 min, 60 °C for 1 min (two cycles), 95 °C for 30 s, 60 °C for 30 s, 68 °C for 30 s (for 27 cycles), and final extension at 68 °C for 4 min. Fluorescently labeled PCR fragments were analyzed on an ABI 3730XL DNA Analyzer, and data were processed using GeneMapper version 3.7 (both from Applied Biosystems, Foster City, CA, USA). UPIC scores, or discriminating power of the markers, were calculated using UPIC software [27].

### 3.3. Genetic Diversity and Cluster Analyses

Considering the low number of individuals by population, we grouped the individuals collected in Mexico in two gene pools: wild and cultivated. Then, to allow comparisons between gene pools with different sample size, the average number of alleles per locus (A) was calculated using the rarefaction method implemented in HP-Rare version 1 [42]. Also, we analyzed genetic diversity for each gene pool using the observed heterozygosity (*H*_O_) and the expected heterozygosity (*H*_E_) with a level of polymorphism of 0.05, using ARLEQUIN version 3.5 [43].

Founder effect due to domestication was estimated with the modified Garza–Williamson index (the number of alleles divided by the allelic range) [43,44], expected to be low in bottlenecked populations, using ARLEQUIN version 3.5 [43]. Also, founder effect was evaluated using the Bottleneck program v1.2.02 [45]. To do this, we performed a Wilcoxon sign test (α = 0.05) to determinate whether a significant number of loci featured heterozygosity excess, which is indicative of a recent bottleneck, assuming two-phase mutation model (TPM. model of microsatellite mutation) and using 1000 permutations.

### 3.4. Genetic Structure and Cluster Analysis

Genetic differentiation among wild and cultivated populations was analyzed using *F*_ST_ and a hierarchical AMOVA analysis [46]. Genetic flow among pairs of populations was estimated with M values (M = 2 Nm) [47]. A Mantel test [48,49] was made to evaluate the hypothesis of isolation by distance. All these analyses were made with ARLEQUIN version 3.5 [43].

The genetic relationships were inferred by three procedures. (1) A Neighbor-Joining (N-J) analysis was made using the standard genetic distance of Nei [50] and the program NTSYSpc version 2.2 (Exeter Software, Setauket, NY, USA). The confidence levels for the topology were assessed by bootstrap resampling (50,000 replicates) [51,52] with WINBOOT [53]. (2) A three-dimensional principal coordinate analysis (3D PCoA) was done using the computer package NTSYS-pc version 2.1 [54]. This technique allows us to explore the genetic structure of the sample data without *a priori* criteria, using each allele as an independent variable. (3) An assignment test of individuals was made with STRUCTURE 2.1 [55] using the admixture model with 200,000 burn-ins and 200,000 iterations to allow the Markov chain to reach stationarity. A total of ten independent simulations were run for each value of K tested, ranging from K = 1 to K = 12. Then, the data generated were used to obtain the ideal K with the method of Evanno [56] using the STRUCTURE HARVESTER program [57]. Bar graphs generated by STRUCTURE for K ideal and that produced considering the sites of collection as different populations were labeled with the drawing software PowerPoint™ 2010 (Microsoft Office).

## 4. Conclusions

It is very important to know about how the domestication process has impacted the extent and distribution of the genetic diversity in vegetatively propagated fruit trees. This work developed and used, for the first time, SSR codominant molecular markers to study this aspect in *P. sapota*, an important Neotropical fruit crop. We showed that the genetic diversity is slightly higher in the cultivated gene pool of *P. sapota* from Mexico in comparison with the wild populations, even though we found evidence of a bottleneck event in the cultivated gene pool, maybe as a result of the domestication process of this species. As suggested by the genetic structure and the cluster analyses performed in this study, wild and cultivated populations of *P. sapota* from Mexico show low genetic differentiation, perhaps, because of the existence of high levels of genetic flow favored by the production and propagation methods of zapote mamey practiced by traditional farmers from Mexico [6]. It is necessary to increase the sample size in order to better understand how the domestication process has impacted the diversity and genetic structure of zapote mamey.

The SSR reported in this work have a great potential for other uses. For example, given the large number of species in banks of germplasm (14,739 plant species in the U.S.A. alone [58]); compared to the number of sequenced plant genomes (approx. 80, [59]), there is a need for developing specific molecular markers to be used to characterize germplasm collections. Also, at the genus level, the taxonomy of *Pouteria* is far from being clearly defined [60]; this genus has a large number of synonyms and its taxonomy is constantly changing [2], probably due to the lack of genetic information. Since microsatellites usually transfer among plant species within a genus [61] and among plant related genera [62], the 368 markers reported here could also help clarify the phylogenetics of the genus *Pouteria*. MegaBlast and BLASTn analysis of the *P. sapota* DNA against the non-redundant database of GenBank (NCBI) for sequences not harboring microsatellites, showed similarity mainly to *V. vinifera* followed by *P. trichocarpa* and *G. max*. The same trend was observed when blasting microsatellite-containing sequences in BLAST2GO (Blast2GO 2009), which uses a different database. Considering that the genus *Pouteria* is non-monophyletic [1,2], the similarity of *Pouteria* (*Sapotacea*) and *Vitaceae* is interesting and should be further explored. Finally, studies have shown the feasibility of elaborating testable hypotheses based on the biological function of genes related to polymorphic microsatellites [63]. Some polymorphic SSR described here had significant hits on BLAST2GO analysis and are worth exploring. Thus, this work is an important contribution, generating a large set of SSR markers for *P. sapota*.

## Supplementary Materials

Supplementary materials can be accessed at: http://www.mdpi.com/1420-3049/20/6/11400/s1.
