# Supplementary material for: Development of a Large Set of Microsatellite Markers in Zapote Mamey (Pouteria sapota (Jacq.) H.E. Moore & Stearn) and Their Potential Use in the Study of the Species
Source: Molecules. 2015 Jun 22;20(6):11400–17. doi: 10.3390/molecules200611400 (PMC6272672; doi:10.3390/molecules200611400)
Supplement: Supplementary file 1 [file molecules-20-11400-s001.pdf]

Supplementary Materials

Table S1. Primer sequences of 368 microsatellites that amplified *Pouteria sapota* samples. ID in bold/italics corresponds to markers that were polymorphic and had significant hits in BLAST2GO. Markers that were not in Hardy–Weinberg equilibrium, observed heterozygosis (H_O) significantly higher ($p \leq 0.005$) than the heterozygosis expected (H_E), are indicated by underscored IDs. Unique pattern informative combinations (UPIC) scores, or discriminating power of the markers [1] are the number of DNA samples discriminated by each marker; only scores different than zero are shown. Allele: Total number of alleles observed for each marker.

Marker ID	Forward (5′ ≥ 3′)	Reverse (5′ ≥ 3′)	Amplicon (bp)	UPIC Score	Alleles
stv-pos_00059_a	TAAAATAAAATGGCAGAACGTGCG	GATAATGGGTGTGGATGGATGG	168–176	-	2
stv-pos_00064_a	TTTTATTCCTTTGGCTTTTCTCGG	GCGTTGAGATCTCCTAAGATTGACC	130–167	1	6
stv-pos_00075_a	AGGAGTCCAAGGAAGAGGAGGAG	TCCCTACCTTAAGATGGACTTTTCTTG	109–122	-	3
stv-pos_00125_a	TCGAATTTCATCAAATGTAGTGCC	ACGGTGGACAATTATATAGTCGGG	172	-	1
stv-pos_00151_a	CCTCTTCCTCCCCTCTTTTCTTC	ACAAGAACCAAATGATGCAGTACG	105–114	-	4
stv-pos_00156_a	CTGCTATTCTCCCTCTCCCTCTTC	TTTAATGATCGCAATTTTGCTTTG	145–158	1	3
stv-pos_00193_b	CAAGGTCTCCTCCTCTCCAAGTC	GAGCCAAAGATGAGAAAGGAAAGG	171–183	-	3
stv-pos_00211_a	TTCATGTCAATCCAACGGTTAATG	CATCAAGTTATGTTTGCCCAAATG	139	-	1
stv-pos_00215_a	CATCATCCATAATCGCAGCTGTAG	CATCGAAAAATTTCAAACACAGAATG	126–128	-	2
stv-pos_00222_a	CAATAACCAACATCTCATCTCGCC	ACGAATGTGTTGTTTTGGGAGAAC	181	-	1
stv-pos_00232_a	CATTTCTGCAACCAAACTTCCTTC	ACATCTTCTTGGGACGATGTGG	143	-	1
stv-pos_00235_a	CCCAGAACTGTTTTGAACTTCACC	GATCCATTTAACCAGGTTCCTTCC	157–160	1	2
stv-pos_00253_a	GCTGATCCTGAACACAAACTCAATC	GTTTCTCTGTTTGTCAGGCTTCG	155	-	1
stv-pos_00271_a	GTTATTAGGGTTTGGGGGACTTTG	TTTCGCTCTGATACCAAGTTGATG	183–185	-	2
stv-pos_00274_a	ATTATCAATTACAAAACCGCGCTC	GTTTCGAGCCACAAGTTCTCTTTC	124–126	-	2
stv-pos_00280_a	TTCGAGTTTCTCACTTTGGTTTCC	AGTCGACTCTGTCGAATTGATGC	162–168	1	3
stv-pos_00286_a	GGTGTGTCCTTTAGGACGTGGTAG	TTTTCTCTCCCAGTTGCTCTTCC	173–186	4	5
stv-pos_00289_a	GACAGGGAGCAGAATTTCATTCC	ATGGAGTTGAAGAGTAGCAGCACC	118	-	1
stv-pos_00291_a	GAAAATTGGGTGTTGGGAAGAAAG	TCAAACTTACCAATGCAAGGTTCC	151–161	-	2
stv-pos_00298_a	ACAACTTTATCATCAAACCACGCC	CTCGATCAGTGTTGGGTTTTCAG	182–186	1	2

Table S1. *Cont.*

Marker ID	Forward (5′ ≥ 3′)	Reverse (5′ ≥ 3′)	Amplicon (bp)	UPIC Score	Alleles
stv-pos_00330_a	AAAATGGAAACCACATAACTCTCCC	GGTTAAAATGGCGGGAAGAAATAG	125–140	1	3
stv-pos_00334_a	GAGATCTCGAACCGAGACGATTAG	ACAAGGGAAACGATCAAGAAGATG	134–145	-	3
stv-pos_00344_a	GTAATGGGGTGGAACTTTCTGTTG	CATATCAAATTCAAACCACTGCTCC	160–163	-	2
stv-pos_00353_a	TTTCTCTCCGACGAGATAGAGAGG	GCACTCCTGGATTCTTTTAGCTTC	123	-	1
stv-pos_00356_a	GCAAGGAAGGAGTACCTTCTAGGG	TCTTCCCTTGAGCGTTGAGTTATC	139	-	1
stv-pos_00356_d	CTTCACTCTCTCCAACCCTAGCAC	CTCGAGCAAGGAACACTCACCTAC	164	-	1
stv-pos_00373_b	CAAACTCTACAACCGAATCCCATC	AGGCAGTGGTTGGACTAACATTTG	144	-	1
stv-pos_00419_a	ACTTCCACAGTCTCTTCCCTTGTG	GCAATCCCTCTTTCTCTCTGTGTC	133	-	2
stv-pos_00423_a	TACCCATTGAGCGCCAAAAAG	TAGGAGAGTGGCAGAGATCAGAGG	101–111	-	3
stv-pos_00438_a	GTAGGTTGTTGTCCAGTGAAAGGG	GTCTTCGTTCTCCCTTTATTTCCG	158	-	1
stv-pos_00450_a	TTCTAGCAAAGTCAATCCACTCCC	ATCTCGCAAATAAGTGTGTGACCC	168–176	1	3
stv-pos_00476_a	TCGATTTCCCTGCTACTCAAATTC	AAAGAACGCGTCTGTCACTCTCTC	154–159	1	2
stv-pos_00489_b	AAGAAATGGTTCAACTCCTAGGGC	TGTTGGTATCACCATGATAGTGGG	130	-	1
stv-pos_00492_a	AACACAATTCCAGAGCCTGAACTC	TTCTATGTTTTGTTGGGTCATTGC	142	-	1
stv-pos_00499_a	GTCCATTTTCCTACCCACAATTCC	GAGAAAGAAAAGAAGGGAGAAAGGG	138	-	1
stv-pos_00500_a	GAGTCAAGTTGAAGACCTTGGAGC	CTCTAATTTGCCATGACAACAACC	167	-	1
stv-pos_00507_a	CATATGATCTGAAGCACCGGAATC	TCTTATGGCTCAGGCTGATTCTTC	167–185	1	4
stv-pos_00518_a	TAAGGGAGAGAAACCTCGTCGAAC	GTGGGGGAAACTTAGAGAAAATCG	180	-	1
stv-pos_00520_a	CTTTCGTGAGGATTTGTTTGATCC	TGAGGTTGTCTAACCTGCCTATCC	142	-	1
stv-pos_00521_a	CTGTTTATCCTCTTTACCCCCACC	GAAGACAAAGACGAGGAAGCAAAG	145–161	1	2
stv-pos_00551_a	TTCTATCCAAACCCCAACTCACTC	ACATTGCCTCAGAGCTCAAGAAAG	179–180	-	2
stv-pos_00553_a	GTGATCTCACAGATTGCTGACCAC	GATGGTCTTTTATGGAGGAGGGAG	130–132	-	2
stv-pos_00565_b	ACTGGGAGAGTTGTGTTCTTCAGC	ATCTCACAGCTTTATCGCGAGTTC	173	-	1
stv-pos_00569_a	GTTCGCAACAACAAAGCCTAATTC	ACGAGTAGGAAAGGGAAAATGACC	144–162	-	3
stv-pos_00582_a	GGCTTATAGGAAAATTTCTTCCCG	TCCCTAGTAGCTACATTGGCTTCG	120	-	1
stv-pos_00588_a	CTCGGTAAAACTCGGCTCAAATAC	CTGACGAGTTCCTCCCTCCTC	146–168	6	8
stv-pos_00594_a	GGTCTTTGTGCACAGTTTGTCAAG	CCAAGTTTCTTAGCAACCAAACAAATAC	138-161	-	4

Table S1. *Cont.*

Marker ID	Forward (5′ ≥ 3′)	Reverse (5′ ≥ 3′)	Amplicon (bp)	UPIC Score	Alleles
stv-pos_00597_a	GAGAACCTCAGCCAGAACTCTCAC	GACTGTTTAGTTTCCTCAATTGGCAC	116	-	1
stv-pos_00599_a	TGGTTCCTCGAGAAGAAAATCTTG	AGCCATAACACTTGTCGCCACTAC	155	-	1
stv-pos_00607_a	AAAAACCAGTGAAGAGAGTGGTGG	ACGGCAGATTGGAACTTCTTACAG	121	-	1
stv-pos_00616_a	CAGATCTGGACCAATTATGGACAG	TGAAGCTCCCAATTTAATCGTTTC	160–169	2	4
stv-pos_00624_a	TTGGATGCCTGCTTTAGGAGTTAG	AAGTGATATATGCTGCAAATGGGG	139	-	1
stv-pos_00626_a	TCCAATATAAGGCTTGGGTGAATC	TTTGAAAGTACTTCCTCACTGGGC	178	-	1
stv-pos_00633_a	CTAGTCATCAGTCTCAGCTCACCG	CTTGGAAAGAGGGGGATGAATATC	154–163	1	3
stv-pos_00637_a	TTCCAGATTTGGGCTTCTTAATCTC	GAACTTCAGAGCACGACCATTACC	106	-	1
stv-pos_00647_a	TCCATATTCCGTTTGTTTTGATCC	ACACAAAAAGATCACCCTCAAACC	181–191	3	5
stv-pos_00661_a	ATCTTTTGGGCATGCTTTATTGAG	AGGTCAGCAAGTTATTGGAAGCTG	168	-	1
stv-pos_00665_a	GCAGCCCAAACCAGTAAGTAAATG	ATTACCAGAAGTGGTGGTCCGAG	93–181	-	2
stv-pos_00669_a	GTGATCAAACAAGGATCTGTGCC	TGCATGTGTCATGCTTGATATTTATG	171	-	1
stv-pos_00679_a	CGTTTCTGGGATTTTCACCATTAG	TTTCAGCTGTTCGATCGAGATTC	168	-	1
stv-pos_00692_a	TTTCTCTCTATCGTCAACCCAAGC	TATGCCATGAACAAGGAGAAGAGG	169	-	1
stv-pos_00708_a	GTGCGTGAGATATTTATGGGGATG	AATCTCCGACTCAATTGACGAGTG	168	-	1
stv-pos_00720_a	GGATTGCTCAGGGACATATGACAG	CACATCTTCAACTCACAAGTTCGC	161	-	1
stv-pos_00723_a	CACCCAAAAAGACAGGAAGAGTTTC	ATAAGAAGATGCGGAGAACTGGTG	126–176	-	4
stv-pos_00727_a	TGAATTCAAGACAAGGAATCATCG	TCATTACTGCCACCACAAATCAAG	180–182	2	2
stv-pos_00733_a	CAAAGTTTCAGGGATCTAGAGGGG	GTGATTGTGGCTTGATGAGTTCAG	132–141	-	3
stv-pos_00754_a	AACTCGTGCTTTAGAAATACCCCC	ATCAAAGGTGAATGGAGTGAAAGG	165	-	1
stv-pos_00756_a	ACCAAGTCCTTCTCCTCTCCAAAC	ACAGGAGAAGAAAGGGAAAACCAG	177–192	3	4
stv-pos_00756_b	ACCAAGTCCTTCTCCTCTCCAAAC	ACAGGAGAAGAAAGGGAAAACCAG	177–191	1	3
stv-pos_00756_c	ACCAAGTCCTTCTCCTCTCCAAAC	ACAGGAGAAGAAAGGGAAAACCAG	177–191	1	3
stv-pos_00758_a	TTTCAAAGATTGGGTTACCTCCAG	TGTGACCAAACAACGATAAAGAGC	118	-	1
stv-pos_00766_a	TTTCTTGTTGTTCCTTTTTGGAGG	AAGCACTCACCTAACTCAACCAGC	117	-	1
stv-pos_00770_a	GAAAACCACTGATGCCGTAGAAAC	CAAAACCGGATCTTGACTTGAATC	141–160	1	3
stv-pos_00787_b	TTTCTTCGTTTCTCTTTGGATTCG	TGCACATGTACAGACACAGCTACAC	184	-	1

Table S1. *Cont.*

Marker ID	Forward (5′ ≥ 3′)	Reverse (5′ ≥ 3′)	Amplicon (bp)	UPIC Score	Alleles
stv-pos_00801_b	TCATAATCATCATCACAACCGTCTTC	CAAACCTTCACCCACAAATTTCAC	182	-	1
stv-pos_00820_a	AGAGACTGGTGAAATTTGTGGTGG	AACAACTCCGGTAGCAAATCCTC	145	-	1
stv-pos_00863_a	TTGACTTTTTGTTTGTTTGATCGG	TTCCCTATCCTTTTTGTAGGGGAC	148	-	1
stv-pos_00871_a	CCGTGGCTAAGGAGAAGGTCTATC	TCTTTGTGAAATGAAAGCAAACCC	132	-	1
stv-pos_00873_a	CAGACTGAAGAAGTTGAGAGTTTGC	TCTGGAAGAATCAGCAATTTCAAG	162–169	-	2
stv-pos_00885_a	TACTCGTTCACCTCTGAGGGATTC	ATGGTAGAACTCACTGGAGGCAAG	154–177	-	2
stv-pos_00901_a	AATTGTGGAAGCAAGGTCATTGAG	TTGTTTGTGCAGTAAATATGCATGG	154	-	1
stv-pos_00906_a	CCAGAGAAAAAGAATTGTTGGAGG	TCCAACGTCTCCTCCTACGTCTAC	142	-	1
stv-pos_00919_a	GAACTGCAAACATAAGCATGGACC	TTTTTATGAAACCTTCCCATGCC	141	-	1
stv-pos_00929_a	GATATTTCCTCCCAACAGGTCCTC	GGAAGCTAATGAACTACGCAAACC	116–124	1	2
stv-pos_00940_a	GCGTGTTTTTGTTACCATTGGTTC	CTTTGCTCATCAGTCATCACCATC	131–136	1	3
stv-pos_00967_a	AGGTAGGGGGTCTTACCAAAGGAC	CAAATTCAAACACTCGCGCAC	175	-	1
stv-pos_00969_a	AACCCTTCATTCCTCAAGATTTCC	ACGTCAAATTGGCAGATTTTGG	179–182	-	2
stv-pos_00971_a	TTCCTATGTCCCTGCAATGTAATG	GATTGGTTCTTTGCTCATGTCAAC	134	-	1
stv-pos_00978_a	CTATAGCGGGGTTACGGAGAGAG	AAGTTGAATAGTTCTCCCACTGCG	180	-	1
stv-pos_00981_a	GATGCAGCTTCAGAGGAGAGTAGG	TAGAAAACTTGCACAACAATTGGC	145	-	1
stv-pos_00983_a	CTGCCATTAAAGAAGCTGGTAACG	ACATTAATGGAAGCAAAGGCAATG	116–122	2	4
stv-pos_00995_a	TCGATTCTGTCCAAAATCAATCTG	AACAAGCAAGTGAAGGAATTTCAAG	176	-	1
stv-pos_01026_a	GATTAACGTCCAAGAAGAACACCC	TCTAGAAGCAACAAAGGACCCAAC	99–103	-	2
stv-pos_01051_a	CTTACAGGAATGGAGAAGTTCAGAGG	CATCCAGAGATGTCCCTTCTTGAG	104	-	1
stv-pos_01056_a	AAGATTCTCTGAAGGCTCATGCAC	AAGCAAAGCTATGGTTTCAACTGC	128	-	1
stv-pos_01060_a	GCGAGAAAAGAGTTCGAAACAAAG	TCACCAGCATCTGTCCTCTTCTAC	147	-	1
stv-pos_01072_a	TCCTGTCCAAGAGGTAATTTCTCG	AAATTCCTTGATACAGGGCACATC	151–155	-	2
stv-pos_01078_a	GAACCACTTACCAAGCTCCAACTC	GGGTATTGGAAGTAAAGAAACCAGG	107–111	-	3
stv-pos_01080_a	TGCACAAATCTGAAAAGACAATGAC	ATGTTGCTAGCATTGCTTGATTTG	133–181	-	3
stv-pos_01086_a	AAAGAAAGTAGGGAAACCGTGAGG	TTTTCAAGAGAGTTTCTCCCCTCC	170	-	1
stv-pos_01092_a	CACTTCAGGGACACCAAATTTCTC	GCAACCTAATCTCCAAACTCAACG	149	-	1

Table S1. *Cont.*

Marker ID	Forward (5′ ≥ 3′)	Reverse (5′ ≥ 3′)	Amplicon (bp)	UPIC Score	Alleles
stv-pos_01096_a	CACTTACTAAGATTTTCTCCTCTCCAAG	GGAGAAAAGAAAGAAAACCGAGAG	170	-	1
stv-pos_01105_b	TGAGTGTTAGGAAGAAGAGCAACTG	CCAAGATTAAGTTCCTTCTAAGCTTTC	113–160	1	2
stv-pos_01111_a	ACATCATGTCCTTGGTCTTCTTCC	CAACTCTTACCAATCGTTTCTGGG	132	-	1
stv-pos_01117_a	AATCAGAGAGAATTGGACGTTTGG	AGAGAGTGAACGATTTTTGTGTGC	122–129	-	4
stv-pos_01124_a	CTCTAGCCAGACAGCTATCCCTTG	TTCTTGCTTTGTCTAATTGTCCCC	170175	-	3
stv-pos_01137_a	AGAGAACCCCACGAATACAAAATG	ACCAAGCAAATAAATAAGCAAGCG	160	-	1
stv-pos_01145_a	TTCCTATGCATGGCTAACGACAC	CCATGTAGGTAAAAATAGGATAAGGCG	121–136	2	4
stv-pos_01148_a	CAACGGAATCACCAAAAGAAAAAC	CTGGGTACTGACAGATTGGGTTTC	155–161	-	2
stv-pos_01157_a	AACCTGGTAAGAAGAAGAAACGCC	GCAAAACCCATTACTTCTGTCTCG	180	-	1
stv-pos_01183_a	AAAGAGACAACAAAAGAAACCCCC	AGTGAATCACCCAAAAGGTGAGAG	128–130	-	2
stv-pos_01184_a	TCAATGGAAAATACAGAATTGGTCG	CTTCGTCTACTGACTGGAGAAGCG	159	-	1
stv-pos_01188_a	TGCACTGGCACTAGAGTCAATAACC	TCGCACCTCCCACTCTTCTACTAC	114–128	-	3
stv-pos_01194_a	GAAGTGCAAATACCATTCCATTTCTC	TGGTTGCTGTGTTCTTTCTGTCTC	168–178	1	2
stv-pos_01207_a	TTCGAAGAAAGAGAGTCCAAGACG	TTCACTTTTCCTTTGCCTTGTCTC	160–167	2	3
stv-pos_01215_b	CACAGCCACCATCTCTTCTTCTTC	TCTCCTCACTTCACTACTCGACGG	116–119	-	2
stv-pos_01225_a	AACCAAAGGTTTCCAGCCTCTTAC	AATTATGCCGAAGAAGCAGATGAG	115–117	-	2
stv-pos_01227_a	ACCGCTACAAGGCAACTACCC	GAGACGTATTTGGAGATGGCAAG	181–225	-	2
stv-pos_01246_a	TGGTAGCTGGAACTTAGTCGGAAG	GTTATGGGACTTGTTAAGGACCCC	113–115	-	2
stv-pos_01267_b	ACTTGCAACCAATAACAGTAGGCG	AAGAAGTAGGTGTTTGACCGCAAC	136–151	1	3
stv-pos_01269_a	CCCCGTACATCTAGTGTGAATTGG	AGGTGGACCAGGTAAGAGTGGAG	153	-	1
stv-pos_01274_a	AACATGCCGAGGAAGAACTACAAG	GACTATTTCTCAACGGTGGCATTC	162	-	1
stv-pos_01283_a	CTTGATGATCTTGTCGAGTGATGG	CAATACCAAGTGAAGGTGAAAATCAG	158–170	-	2
stv-pos_01307_a	ATTTGTGTCCTCTCTTTCACCTCG	ATGGCAATCTACACACAAACATGC	174–176	-	2
stv-pos_01316_a	GCTGTTGTCCTCTTTACCCATCAC	TCAACAGGGTGAAAGAAGCAGAAG	105–216	2	5
stv-pos_01328_a	CCTTCAACTGTTCTTTTCCCATTC	TTCAAAGGAGGTTCAGACGAAGAC	176–180	1	3
stv-pos_01332_a	TCAAATACCATGAATGACCGAATTG	GCAGTGGTTAAGAGGCTTGATGAG	167–180	1	3
stv-pos_01351_a	TTTTGTTCCTACTTCGGTAATACGTG	AATAAAGTGAAGCTTCCCATCCAG	122–124	-	2

Table S1. *Cont.*

Marker ID	Forward (5′ ≥ 3′)	Reverse (5′ ≥ 3′)	Amplicon (bp)	UPIC Score	Alleles
stv-pos_01359_a	TAAAGGATGCAGATCAAGAACACG	AAACCCTTTCTTGAACCTCTGGTC	121–124	-	3
stv-pos_01364_a	GAAATTCTTGGAGGACAGGTGTTG	TTCAATCTCCTTCACCCCTTTTTC	151–154	-	2
stv-pos_01380_a	CGTGACATACCAGTAGCTTGTTGAG	GTGGTGATAACGACGACGATG	127–217	1	3
stv-pos_01387_a	CCTCTCCCTGCAAACATAGTTGTC	TTTCATAGCCCTCAAACCAGAAAC	128–130	-	2
stv-pos_01402_a	GCTAGTTTGAGAGCCATGACTTTTG	ATATCACACCTCAACCTCGCTCTC	147–156	-	3
stv-pos_01436_a	ATCGACTGAGCAACACCAAAATG	GATCCAACCTTCTTCGTTTCTTCC	163–166	-	3
stv-pos_01438_a	CCTCATTTTGTATTTGCTTTTGTGG	AATACGGCTTTATTCCCAAGACAAG	173–175	1	2
stv-pos_01441_a	TTCATAATCCAAGGCATCTTCAGC	TTCTAACTCTGGCGGATCTCAAAG	167	-	1
stv-pos_01450_a	CCGGCAATTACAACTTGAGCTATC	TGACAGAAGTAAGAGACCTTCTGCG	155–161	2	5
stv-pos_01452_a	GATTTTCAGCATTGAACCCATTG	ACACGTCAAAAACCAAGATCCTTC	168–174	-	2
stv-pos_01463_a	GATCCTTCAAGCTCGACAAACAAC	CCCATCGAAAGCCTCGACTAC	171–172	1	2
stv-pos_01463_b	ACTCTTCTTCGTCAACAGTCATGG	CAATTGAAATTGAACGGAGTATGC	119–125	1	3
stv-pos_01465_a	AAATCACACAAGTCACACATGCAC	TTTAATTGGAGAGTCGCTCTCAGG	137–138	2	2
stv-pos_01475_a	AGTCGACCCCCTTTAAAAACTTTG	TGAGAAGGGTGGTCTTTGTTGTTAC	159	-	1
stv-pos_01499_a	CGATGCTTTGTTTTCTCCTAATGC	CGACCTTAGAGGATGAACCATACG	176–186	1	4
stv-pos_01505_a	CGTCACCAGCACAATCATCTTC	AGACATTTCTGCAGGTGAAGGC	96–223	10	17
stv-pos_01512_a	GATAAGGTGTCGTCTAGGTCTCCG	TTTCTCACTTTCGCGGTAAGTCTC	147–185	6	7
stv-pos_01520_a	GAATCCTCATGTTCCCTTTCTCTG	CTCCAAAGCCACCCTTTTATTTTC	116–131	-	3
stv-pos_01523_a	AGACGGCGAGATAGATTTCAAGTG	TGTAGCTCTTCGTGTCAACTCCAC	133	-	1
stv-pos_01529_a	CTGAACAGCTGCTTTGGATTCAC	TCCTCCTTCCACCTCAAACCTAAC	163	-	1
stv-pos_01539_a	CGAATCTGATGACTCGTCTTCCTC	TGCTCTCAATACCACTGAAACGAC	161–165	-	2
stv-pos_01567_a	TTCTTCACCTCTTCCAAGTCCAAC	CAGTACAGATTTGAGGCCACTGC	140	-	1
stv-pos_01578_a	GATTGGACGGAGAAGAATACATCG	ATACTTTCAGTGACAGCCCCACC	162–196	-	2
stv-pos_01580_a	GGGAGAGCTTGAAATTCCCTGTAG	TCAAGTTCAAACTTCCTCCGTTTC	172–186	3	6
stv-pos_01583_a	TCCACCATTTTCCTTCTCATTTTC	TGATGTCGTTGAAGTTGGAGTAGC	171	-	1
stv-pos_01587_a	TTCACTATCAGGGAAGCAAAGGAC	TTGCAAACTGCAGGCTGTTAATAC	172	-	1
stv-pos_01589_a	AGTCCATTGGATCACGTCGAATAC	AGAATGAAGCGGAGAGAAGAACAG	170	-	1

Table S1. *Cont.*

Marker ID	Forward (5′ ≥ 3′)	Reverse (5′ ≥ 3′)	Amplicon (bp)	UPIC Score	Alleles
stv-pos_01602_a	ACTTCAAGAACCAAAAGCAAAAGC	AGTTGGTGTTTACTCAAGCTGCTG	172–188	-	3
stv-pos_01609_a	ACGCATAGCCTATTTTTCTTGCTG	AGAGCAAGGAAGGAGTAAAGGAGG	177	-	1
stv-pos_01612_a	ATAACATTTGCTGGAGTCTTTGGC	TGATCAGCTTAGTCAAATCACATTACC	170–171	-	2
stv-pos_01628_a	ATTCTTTCTCTTCACCTCTGCACC	TTTAGTTTTGGACTTGAACGGAGG	104–108	1	3
stv-pos_01632_a	AATCAACTTTGAATCAACCGATCC	AATGCGCTGCAATCTGTAATTTG	179–184	2	3
stv-pos_01635_a	AACACTTGATGTGAGAGCACTTGG	CCACCTACACCAATACCATTCCTTC	155–159	1	2
stv-pos_01640_a	GTGCGTGGATGCAATTAAGAAAG	AATAATATAAGCAAGCCCCTTGCAC	145	-	1
stv-pos_01652_a	AATCCTTTCGCTCTCTGTTGTTTC	GTTCATTATGGCAGAAGTCACAGC	153–156	1	2
stv-pos_01656_a	GAGAAAGAGCTGAAAAAGCTGCTG	CTTTACGCCGTGCTTCAGATTC	131	-	1
stv-pos_01673_a	GAAACTCTTTGGAGTGAGAGCGAG	TCTCATTCCTGATTTCTTCCAAGC	144	-	1
stv-pos_01675_a	GAGACACCTTGCATGTGCTTAGAG	GGAGACCTTGGTTTGTTAAACGAG	140–159	3	5
stv-pos_01677_a	CTCATTGTTTTTCACGGTCACTTG	AACCGTTGAAGTCTACTGCCTCTG	171–179	3	5
stv-pos_01685_a	GGGAAGGCATTTTACCATCAAAAC	CCTCCCCCTATTCTTCTTTCTTCC	181	-	1
stv-pos_01688_a	CTGCTGATGGTTCTTGATTGTGAG	AAGCTTCTCGAGGAGAACGGTAG	104–113	-	2
stv-pos_01694_a	GCGATATGGGTCGAGGACTG	GAGAGTAGAAGTGACAGTTCGTCCG	122–126	1	2
stv-pos_01696_a	CCAAATGAGAAATTAATCCCAAAAC	GAACCCAAATTAGTTTGTTAGTATCCTG	176	-	1
stv-pos_01722_a	CCTCCATCCTCCAGTAACACCTC	GTAGGAAGAGGAAGAAGGTGGTGG	143–160	-	2
stv-pos_01732_a	AACAGAGCTTTTGGACCTTCAATG	TAAGATAGGGTCTCCTTCCACTCG	108–110	1	2
stv-pos_01755_a	AGAAACTGAAGGGGATGGAGAGAC	GCAAATTGGTCAGTTTGTTTTCATC	171–177	-	2
stv-pos_01769_a	CCACCTGTCATCTTCCTCTTCTTC	CTTGTGCCAAGTTTGAGAAGTTCC	151	-	1
stv-pos_01779_a	TGATAGTGCTAAGGCTAAGATCAGAAC	TCTTAAACCACTCTTTCCTCCTCC	108–138	1	4
stv-pos_01788_a	TATAGCTCCACACATAGCCGTCTG	AAAAGCTGGTTCTGCGTTACAAAG	159	-	1
stv-pos_01791_a	TTGTTCCTAGTGCTGGAAGAAACC	TGTCCTTTTCTTGTGGATTATTCTCTC	175–182	-	2
stv-pos_01799_a	TGACCCTGAAATTTATGATTCTTCG	CGAGATGTACCATTTCGTCATACTG	180	-	1
stv-pos_01801_a	GGCATATATCTGGAAGGCTTCATC	ACCCATGTTTTCAACACAGAACAC	162	-	1
stv-pos_01812_a	TAATTCAAACCGAGACCTCATTCG	CAGTCGGAACTGCGAAATCC	120–266	1	4
stv-pos_01818_a	GTTTTTGGTGAGGGATTGTTTTTG	ATAAATTTGCATGTTTGGGTCTGG	158	-	1

Table S1. *Cont.*

Marker ID	Forward (5′ ≥ 3′)	Reverse (5′ ≥ 3′)	Amplicon (bp)	UPIC Score	Alleles
stv-pos_01823_a	TGAGAAGTGAAATGAGACAGTCGG	CAACCCATCCATTTGAAGTAATCG	179	-	1
stv-pos_01827_a	TCAACCAATAAGGGTCAGGTTTTG	GATCAAATTGGCACTTAGAGCACC	123–181	-	2
stv-pos_01837_a	CTCCGTAACGCTTCCCTTTACTTC	TTTCTTTGCCATTAGTTTGTTCCG	173–182	-	3
stv-pos_01840_b	ACTTTCATCACCACCACTCCTCTC	AGCTTTAAGCGTCCGTTACTTGTG	168–180	1	3
stv-pos_01857_a	ACCGAAGATATGGTAGTGGTGGTG	TTACCAAAAGGATTTATCTCCCGC	155–180	-	3
stv-pos_01865_a	TTATGTTTGGGCTTTTGGCAATAC	GCTGGCTCATTGACACCTCTATAAC	167	-	1
stv-pos_01867_a	AAAAATGCTCTGGCAACAGAGTG	ATACAGAGAGGCAGACCTCAGCAG	176–181	1	2
stv-pos_01868_a	TAAGACAGTGTCCTCTTTTTGGGC	CCCACAAAGAAAAGAATGACAACC	184	-	1
stv-pos_01876_a	GAGCAAGAGGGTTAGTGAGTGAGC	CAATGAAAAACGCTATCTGTGTGC	157–161	1	2
stv-pos_01877_b	TCCTCCTATCATCATCCTTTACCC	AAATAGGAGAGCAGGGACAGAGTG	144–184	4	6
stv-pos_01885_a	GAGTCGAGGAAGAGAACCCATACC	GACAGTTGTCAATTGATGGTGGTG	250–260	2	4
stv-pos_01906_a	GTAATCGAAGTCCATTTCGTCGTC	TTAAGACCCTAATGGTTTGGGAGG	168	-	1
stv-pos_01907_a	GTTGTCGTTTGTCGTTCCTAGTCC	TGTGTGTAAATTTTAAGGGGCTGG	182–184	1	2
stv-pos_01920_a	AGGACAATGATGGTGAAGAAGAGG	TTTTTACTTCTCTTCTTTGGGGGC	134–137	-	2
stv-pos_01922_a	AGAATCACCACTTCTATCACCCCC	CTAACACGACCTGCAATTACCCTC	178–194	1	3
stv-pos_01930_a	GTGTCTGTGGTCAGAACCGTAGTG	GCAGAGAATACTACCCCCAAGAGC	162	-	1
stv-pos_01934_a	AAAACAGCAACTGGATCCAACC	GCTGTGCATTTGTGAGCTACTTTAG	170–174	-	2
stv-pos_01947_b	TCTGTTTCTATGAAGACCCACTTCG	AAGGAGAGAAGAAGCAAGAGGGAG	181	-	1
stv-pos_01955_a	CTTCAGCAATGTCTCATGTTAGCC	AGACACCCTTTCTTAGCCCCATAC	122–294	4	5
stv-pos_01959_a	TTGGTGATCCAGAGACTGAATACG	AAACGAATGTATAGAGGTCCAGGG	164–176	-	3
stv-pos_01963_a	GAAATAGTAGGCGAGCGTCAAAAG	TGTAGGGGAGTAGAGGTTACTGGC	154	-	1
stv-pos_01967_a	TACACGACGAGGAAGGAGAAGAAG	AAGCTGCATTAGCTCACTTGGTTC	105-179	1	4
stv-pos_01968_a	ACCCACTCCCCAGAAAATATATCAC	AAATAGGAGAAAGGAGGTGGCAAG	144–151	-	2
stv-pos_01970_a	CCATGTACTGTAACTTGACCACGC	CGTCGTCAAAGAATCCTCTTCTTC	115	-	1
stv-pos_01982_a	ATCTCAAGCGAAGAATTGCAAAAG	ATCTTCCTCCTCATCCTCTTCCTC	155–174	2	4
stv-pos_02002_a	CTCCTTTAACATGTCTCCGTTGTG	CGAGAACAATTGTAAAAAGAAAACAGC	146–167	1	2
stv-pos_02010_a	TTGCCCTACCTATCAACCAATCAG	GAGTGCTTTGTTGAGTCTTTGCTG	172	-	1

Table S1. *Cont.*

Marker ID	Forward (5′ ≥ 3′)	Reverse (5′ ≥ 3′)	Amplicon (bp)	UPIC Score	Alleles
stv-pos_02013_a	AGCAATTTGATAAGCAGCAGAAGC	CTGACAAAGAGGAGTTGGCAGAAG	154–166	3	4
stv-pos_02016_a	CCAGCTATCAAGGCTACTTTAGCG	TGATGTACCAGCATCTTAGCAAGG	133	-	1
stv-pos_02017_a	ATGGAACGAAGACCAGAGAGACAG	GAAGGGCTCTTGATCCTCGAC	99	-	1
stv-pos_02036_a	TTGAGGAAGGTTTAGTGATCGAGG	CTCTACGACTGCTGTTCTTCTCCC	150	-	1
stv-pos_02051_a	GAGACAACGATTTTCAATGCTTCC	TTCCAAAAATCAGATCCTCGTTTC	152–182	5	5
stv-pos_02074_a	TCAGGCAGGAAGGTATAAGACAAC	CATGAGGACTGCTAAAGTAGTGGC	250	-	1
stv-pos_02091_a	TCTGCAGCTTTGCAGTTGTTATTC	CAGCAAGGTTAATTGCTTCATTCC	150–156	-	3
stv-pos_02095_a	ACTCCTTCTACGTGTCCTCCTCTC	TTTTGATGTTAGATTGGTTTATGGATG	154	-	1
stv-pos_02101_a	AATTGAAAACTGTCTGCCGTTAGC	GGATTTGTCACTACCAACTCCAGG	181	-	1
stv-pos_02114_a	TTGTCCTACAGAGACCAGAGGGAG	TCCATGCTGTGTAATTCTGAATCC	96–121	-	3
stv-pos_02115_a	GAGAAATGTTCCAGTGGGAGGAG	ATAGGTTAAAGGTGGGAGAGTCGG	177–202	-	2
stv-pos_02116_a	CTGGTTGTGAATATTTGATTGATGATG	GTGGATGCAGAAAGAACAAGAAGG	112–172	-	3
stv-pos_02134_a	GAGCTGTCAGATACAAATCCTGGG	GACGGTGATTATGGAATGTGTGAC	160–169	2	4
stv-pos_02137_a	GCCTCGAATAGCTAGAGCAGTCTC	CCCTGTATATTTCGTCACCTCACC	158–162	1	3
stv-pos_02145_a	TGCTTCCTCTTCACCCAACTCTAC	GAACCCTACCTTTAGCTTTGCTCG	167	-	1
stv-pos_02153_a	AAAATTGGAAAAGTGAGACCATGC	AAGAGTCAAACCAAATGCAACTCC	94–115	1	2
stv-pos_02154_a	ACCGCATCAACATCCACATTC	CAAAACCGATTGCAAATTATACGG	183	-	1
stv-pos_02162_a	ATTCCCCTTGTCCAACAATCATC	GGTTTTTCCATCAATGTCTTGTCC	124–127	-	2
stv-pos_02175_a	CAAATTGCTAAAGCATTACTCAGGAAG	TTCAACAACTCAAAGGAACTGAGTG	100–177	-	5
stv-pos_02179_a	GCTGCGACAACACAACTCATAAAC	AAGTTCCTGCAATCAACACATGC	162–170	1	3
stv-pos_02184_a	TATTCAGGTGAAGTTTCTGCTCCG	ATACCTCGCTTGTCCAGCAGTATC	153–157	-	2
stv-pos_02192_a	TAGAGAATTCGCTGGAGGAAGATG	GATAAAATTGGATCGACCGTCAAG	157	-	1
stv-pos_02193_a	TCTCTTTCTCTTCAGCTGACTTCTCC	TTCAGAAAACGAAGAAACAGAGGC	119	-	1
stv-pos_02219_a	TGATAATGTTCTCAAAGATCTGCCAC	TGGAACATGTCATTTGGATTTTTG	118–126	-	2
stv-pos_02221_a	AAATGATGCATTGTCCATATTCCC	TATTCTCTCTGCCTTGCTACACCC	122–143	4	5
stv-pos_02225_a	GTGTTTCTTTTGACGTGTTCGTTG	TAGATACTTGACCGACCCCAACTC	161–165	1	3
stv-pos_02269_a	CTCCACACCTCCGGTCATATTTAG	GATTTTGGCAATCCATGAAAGTTG	141–161	1	5

Table S1. *Cont.*

Marker ID	Forward (5′ ≥ 3′)	Reverse (5′ ≥ 3′)	Amplicon (bp)	UPIC Score	Alleles
stv-pos_02270_a	CAGGAACGTTTCAATTACGGTTTC	GGTGCGACTAATGCAGAGATGAG	170	-	1
stv-pos_02282_a	GATAATTTCAACCCCAATACACGC	GCACAAATAACAAGGGAACCAAAC	168–172	1	2
stv-pos_02287_a	AGTTTCAACTGCTTCATCACTGG	TTCACATAACGTTCTCCAGTTGTTATAC	170	-	1
stv-pos_02304_a	AGTGTTCTTCGGGTCTTGATTGAG	CCAGTGCAGAGGACGGAGATATAG	104–114	2	3
stv-pos_02323_a	CACACAACAACTGGACACCACAG	CGGGTTCTCAAATACAAGAGATGG	178	-	1
stv-pos_02329_a	AGATATCCCTCCCCTGGTAAACAC	GGTACGAAGCACAGTAGTTTAATGAGTG	123–138	-	3
stv-pos_02335_a	ACACAGATTGCGTGCCGTTAC	TTTCCATTGCTTTTCTCTCTTTGC	175	-	1
stv-pos_02338_a	AATGACAATCGGGTGAACAGAGAG	CCTTAGACGACCGCTTTTTCTACG	176–178	2	3
stv-pos_02340_a	CGGGATTGAAAGTAGTAAGGCAAG	TCAAACAAACAGAGTCATAAAAAGGG	146–148	-	2
stv-pos_02343_a	GCTCGAAACCCTAGTTCGATTCTC	TTTACCTGAGAAACTGTCTCCTCCC	108	-	1
stv-pos_02349_a	CCTCCTCACTCACACAAGAAGACC	AGAAGCAACAAACTTGTCGAGGAC	138	-	1
stv-pos_02355_a	TGATTTGCAGCTGTAAGAAACACC	GGAGTCCAGAAAGTTACCACAAAGAG	136	-	1
stv-pos_02365_a	TTCTGAGTGAGCAATGGTTTAGCC	CACAGAAAAGTCTTGTGATGTGGG	174–182	1	3
stv-pos_02374_a	CCACCTCCTTTTTCTTCTGATCTC	CGGCGTAACCTCTTTCAAGTAATC	159–161	-	2
stv-pos_02375_a	AAATATGACTCCTGGCTCGAGTTG	AGCGCAGTTCCTAATTTCTGTCAC	157–166	1	4
stv-pos_02378_a	TTGGATTTGGGGAAGAAGTTTACC	CATGGAGGATACTCTGCCTTTTTG	167	-	1
stv-pos_02388_a	TGGTTTGGTAGTTATAGGGGATTCG	GTAGTAGCGGCAACCCGAATTATC	172–174	-	2
stv-pos_02398_a	ACGAGAGTAAAGCAGTAGGCGAAG	CTTGCTTTCTATCACCTCCGTCAC	183	-	1
stv-pos_02408_a	GATCGGAAGCTCCAGTTCTTCAC	CTGGACCCATTGAAGAATTACTCG	105–108	-	2
stv-pos_02417_a	GTTGATTACTTCAATGGAGGGGTG	TCTATTGGCAACATTATGGGATTG	178–182	-	3
stv-pos_02419_a	ACTATGGTGCTCCAACCAGCTATC	GACCACCATAATGAGGTGACTGTG	109	-	1
stv-pos_02428_a	TCTGTCTATGGCGAAAGAAACAAAG	ACCCCTCTTCAAAATTTCGTCTTC	161	-	1
stv-pos_02453_a	TGTTCCTATCAAGCCAAAAATAAATACC	GGGTTGACATTGCTGAAGACTACC	162	-	1
stv-pos_02461_a	TTGTTGTGGAGTATGTTCCCAATG	CATGCCCACAGCTACATACAAATG	171–179	1	4
stv-pos_02469_a	AGAGCACTCATGTGCTTCAGTTTG	CTCCAACATTCTCACTCCCCTC	118	-	1
stv-pos_02473_a	TGGTTTTTCTACTTGATCTTTTGCTTC	GCCACTCTCTTGATTCTAACCCAC	164–166	-	2
stv-pos_02479_a	CATCATCTAATTCCCAAAACAAACG	CACCTTGTTGGTTCATCCTTACG	130–135	1	3

Table S1. *Cont.*

Marker ID	Forward (5′ ≥ 3′)	Reverse (5′ ≥ 3′)	Amplicon (bp)	UPIC Score	Alleles
stv-pos_02484_a	AAGGATAAGAAGTCCCATTGGCTG	AAGATTAGAAGGGAGCAGCAGAGG	173–182	1	4
stv-pos_02486_a	ACGCTTTGTTTGTTGCTCAGAGTC	CAGAAGAACCAGATCTATGTCGCC	126–129	-	2
stv-pos_02490_a	AGCTAGGATCACATCACATCTAAACTTG	AGTCTCTGGCAACTTTTTGTCCTG	152–193	4	5
stv-pos_02496_a	TGCAAAACTCTGTGCTAGTTGATTTC	ATATTCAAAGACTTTTGGGGAGGG	126–137	-	3
stv-pos_02515_a	CAGAAAATTCTCCTCTACGCTTGC	GAGATTGGAGCAAATGTGACTGTG	168	-	1
stv-pos_02535_b	TGAGATGAGAAGCACCAAACAAAC	CTGTTTCCCGTCTTTGCGTTAC	146–153	-	4
stv-pos_02565_a	CCGAGAAAGGGATAGAAAATGGAG	AGAGAGTTACCCCAATCCAAAACC	157–159	-	2
stv-pos_02570_b	ATCCATGGAGGTGTTTGATGTTTC	CTAGCTAGCTCGACCCTCAACAAC	153–157	1	3
stv-pos_02582_a	CATCTCTAACTCTTTAATCTCCCTCCC	ACGACCAAGATCATTCCTCAGAAG	166–170	-	2
stv-pos_02588_b	CAGTAATCCAAAGTGATCCAACCC	GGTAGTCACTGGCTTGATTGTGC	123–290	1	5
stv-pos_02590_a	AGCTGGACATTGACATGGACATC	TCGGATTTGTTCGAGATAGAGAGC	127–130	-	2
stv-pos_02592_a	CGATAATTGGCACTATTCTTACGG	CATTAATTGGATGATTAATTTGGAGG	168	-	1
stv-pos_02596_a	ACAGAGCCAATCTTGGAGAGACAG	TTCCAAGAACCAATCAAGAAAACC	162	-	1
stv-pos_02604_a	ATTTCATGCAACAATTCCCTCTTC	GTACATGTTCCTTTGGGTCTTTGG	158–160	-	2
stv-pos_02605_a	ATACAAAAACTGCCAACTGCCAAC	TTTCCTCTCTATGGCATGATTTGC	182–188	1	2
stv-pos_02614_a	TGAGTCTGATTTTCTCGATCGTTG	CTGGTCGATTGTAAACACCTACCC	176	-	1
stv-pos_02634_a	CATGAAACATAAACATCTCACCAAGC	TGAACTATCAGAATTGGAGGTGGC	162–164	1	2
stv-pos_02644_b	TGGGAGAAAGTCTAACCTGGTTTTC	ATCTTGTTTCCTTCGCTACCCATC	112–128	4	6
stv-pos_02654_a	GATGGATTTGAAGCATTCAACAAG	ACAATTTCCCCAAATTTACACAGG	180	-	1
stv-pos_02660_a	GACTAATCGGTCTCACAGAGAGGG	GTTCCAATTTTCTTTCCCCAACTC	152–158	3	3
stv-pos_02670_a	AGGGTTCTTATGACCTTTGCATTC	TGGACAGATGGATGTTTCTATGTATG	146–155	-	3
stv-pos_02674_a	AATGACAAAACAACCAAGAGGTCC	TTTGGACAGACCTCTCCACTTCTC	134–142	1	3
stv-pos_02695_a	AAGTTATAGCGCCACTGCTTTCAC	GGAACTCTGATGACCAAGAAAACG	169	-	1
stv-pos_02709_a	TCTCTAGACGAGGATGCATTTCAAG	TGTGGTGTCAACATTCCCTACAAG	139–144	1	2
stv-pos_02722_a	TCTCCTCTCCGATTTTATCTGCAC	AAACGGAACCATCACCTTCATTC	168	-	1
stv-pos_02726_a	GTTATTCTTGCTAGGTGTCTCGGG	ACTTCTCCAAACCCTTCTTGATCC	134	-	1
stv-pos_02728_a	AAGGAATGAGCTTGTGTCCTTTTG	CAGTTGCCTTTCTTCCCAATACC	173	-	1

Table S1. *Cont.*

Marker ID	Forward (5′ ≥ 3′)	Reverse (5′ ≥ 3′)	Amplicon (bp)	UPIC Score	Alleles
stv-pos_02730_a	TCCATCTCATCATTCCTCAATTCC	GATCGATCATCAAGGTTGGAGTG	172	-	1
stv-pos_02743_a	TGTGCAGAGCCGAGATTAGTACAG	CAACCAAAACTCCAAAACCATGTC	113–176	-	4
stv-pos_02748_a	CAGAGGTCCAGAGGCTTAGACAAG	CATCGCAAGAGGTAGAGATGAGTG	176–194	1	3
stv-pos_02755_b	TGTAACAGTTTTCAAAATTTCACGTC	TGGTGAATATGTAATCATTGCAAAATAG	126–143	3	5
stv-pos_02756_a	TTGAGGTGAGGAACAAACCAATTC	TTGGAACCACCTAATCACACATTG	101–103	-	2
stv-pos_02758_a	GGGAATTTCTCCGTTCAGGC	CGTCTCCCCTTCCAAGTAGAAAAC	180	-	1
stv-pos_02768_a	AATTTGTACCTGGAAAGCTGTTGTG	CATCATGACAGGAAAATAACAGGG	130–135	1	3
stv-pos_02769_b	CTACTAATGGAAGGCTTGGCAGTG	AACACTCCTTTCCTCCATCCAAAC	150	-	1
stv-pos_02771_c	CCTTTGCCTTTGTTCTTTTGTTTG	CGGTTACACTCCCTTTTGTGTCTC	166	-	1
stv-pos_02781_a	AAAAACTGGGTGCCACGTTATTAG	TGATTAAACATAAATAAACAGACCGGG	153–177	1	4
stv-pos_02782_a	AAGATAAGAATTGCCAAACCCCAG	AATACCGTTTCCGGTGGAAGATAC	179	-	1
stv-pos_02788_a	GCCTGTCTTTGTTAGAGGTTTTGC	AGGAGTACAAGAAAAGCAGGATGC	163	-	1
stv-pos_02791_a	AAAATCAGATAACATTAGAACAATGCTG	GTTGAAGGAATGCAAGGATTAAAG	180–238	2	7
stv-pos_02799_b	GAATAAATGCACATGATTGCCAAC	CGTCAAGTCATTGAATGTGAAGATTAG	175	-	1
stv-pos_02807_a	TTTGCGAGGTCTTCCTCACTTATC	CATCGTAATGATGTTGCTGAGGAG	168	-	1
stv-pos_02813_a	TGCACAGTAACAAACCCATTTCAG	TATATTCTGCTGGCTCTGTTTGG	167–169	-	2
stv-pos_02822_a	CTAAGCCAGCCAACAAAAACTCAG	ACCTTTCTCAATGCAGTTTTCTTCTC	161–163	1	2
stv-pos_02857_a	CCCATGAGATTATCAATTTGGTCC	CAACATATGAAGAAGAAAAGGGGG	169	-	1
stv-pos_02858_a	AATATTATCGACCACCATCAACCG	GTGGTCGTGGTAGTTAGAAGTGGG	167	-	1
stv-pos_02862_a	TTCTCCGTATCTTCCATGATTTCC	TACGTTCTACGTGGCATTCAACAC	156–173	3	5
stv-pos_02874_a	AAGTAAAATTGCCATTGCAGTGAAG	CTTCCTTACTTCTCAACCGAGCG	166–172	1	3
stv-pos_02878_a	GCGGTTTAACACTTGAACAATTCC	CATTGGTCATTGATGAATTTATTTGG	142–157	1	2
stv-pos_02879_a	AGGTGAGGGGAAGGAGATGATG	TCTCCTCAGTATTCTCCACCGAAG	147	-	1
stv-pos_02881_a	CAAATCATCCCTTTTTCACTTTGG	ATGCTAGCTCTTAGAGGGCAACAC	129-132	1	4
stv-pos_02918_b	GATCCAATTAGCAAGCGACAACTC	CAATTCAACTGAGGATTTGGACAAG	138	-	1
stv-pos_02934_a	CAAAGGGAAGAGCAAAAGTGAAAG	CATCATTTGATTCATTGGAAGAGG	177–178	-	2
stv-pos_02938_a	GTCGGTTGTTAGCTCTGTCTCTG	GCAGGCAGACAATTAATTAAAAAGG	184	-	1

Table S1. *Cont.*

Marker ID	Forward (5′ ≥ 3′)	Reverse (5′ ≥ 3′)	Amplicon (bp)	UPIC Score	Alleles
stv-pos_02954_a	ACCTGGTGCATATGGTTAAATTGG	TCTACCATCTCCAGCGTAAGTGTG	134	-	1
stv-pos_02962_a	ATTATTACCACCACTTGTCACCCC	TGATGAAATTTGAAGGATGCTCTG	163–169	-	2
stv-pos_02963_a	ACGATGAACCAGTTTGCTTTCTTC	TGTTCTACGATTCCATCATCAAGC	110	-	1
stv-pos_02977_a	AACAGAAGCAATGAAATGTCGACTC	GGCTCTGCTTTGATCTTCTTCTTC	172–174	-	2
stv-pos_02987_a	TCCTCACACAAGAGGAAGAGAGATG	CACCTCTTCCATGAATATGAACCC	175–179	-	2
stv-pos_02994_a	TAAGTTCTTGCACGCCTGATTATG	GTATGGTATCCTACATTGCCTGGG	171–175	1	2
stv-pos_02997_b	ATTCCCGCTGATTTGGTTTAATG	CAACAACTCTCCAATCTTCAACCC	143	-	1
stv-pos_03102_a	CAAAGTGCAGAAGAATAAATGGGG	CTGACCTTTCCAGACCCTACAATC	164	-	1
stv-pos_03146_a	CAACAGTGTCCAACCAATTCTCTC	GATCCAACAGCAACCCTATATATCAAC	164	-	1
stv-pos_03228_a	AGCAGCCATTAATTTCCCTTTTTC	GCTGGTCTCTGTGCTACCTGTTG	158–161	-	2
stv-pos_03233_a	GGTTTGGATGGTGACAATCTGAG	CTGTGCGAGCACTTGGTCTACTAC	180–184	-	2
stv-pos_03247_a	GTACAAATGGCAGTGTGCTGTTG	AGAAAAGGGCAGGTGTAGAAGAGG	157	-	1
stv-pos_03248_a	TGGGTCAAAGGAGAGCAATTTAAG	GTCCATCCTATGCTGTTGTTTCTG	168–175	-	2
stv-pos_03300_a	CACCAAATTAGGGCTTTCACAATC	GTACGTTCGACCGTTTTCGC	111	-	1
stv-pos_03313_a	TTCACTCTTTTCCGGAATCAAAAG	GTTTTCAACCTCCAATGTCAATCC	171–180	1	2
stv-pos_03329_a	TTGTTGCTTGGTTTTTCATTGTTG	GTGGCTTGTTCTCCCTCTCTGTC	165	-	1
stv-pos_03331_a	AGCATTTAAGACACAGCACAGCAG	TCTCTCTAGAAAACGGCAAAAAGC	149–161	-	3
stv-pos_03370_a	AAATCTCCACGTAACCGTTCTGAC	GAAAGAAGGGAGGCGATGATG	155	-	1
stv-pos_03382_a	AATAGCATGAACACCCCTCGTATC	ACCATAAAGCTCTTGGGAAGTGAG	160	-	1
stv-pos_03392_a	GGACAAAGAGGAAAGCAACAAAAG	GATGATCACATTACAGGACAACGC	135	-	1
stv-pos_03526_a	GGAGAGGAAGTGATGTAGGACGAC	CTCCCCTCTTCTTCTTTCTTCCC	119	-	1
stv-pos_03536_a	AATAGTCATAGCTCGCTCACTGGC	ATAAATACCAGCCAACCCTCTTCC	140	-	1
stv-pos_03545_a	TACGTCGTCGAAGAAGCTATCGAG	ATTCACATGGACGACATTGAAGC	103	-	1
stv-pos_03555_a	GAAATGATTCTCTCTGGGCAAGAC	AAAATCCCATGTCCAAACAACAAC	138	-	1
stv-pos_03564_a	AATCCTGCAACAATCCAGAAGAAC	GGAATTCCTTAACCGTAGCCAATC	136	-	1
stv-pos_03569_a	ATAACATTTCCATCCCTCCCAATC	TTATTGTTCAAGATGATGATGCGG	144–162	2	4
stv-pos_03597_a	CTTCATGTTTGCATCGTTTTGTAG	TGTAGAAAATACCAGATATGAGAAGACG	154–161	-	2

Table S1. *Cont.*

Marker ID	Forward (5′ ≥ 3′)	Reverse (5′ ≥ 3′)	Amplicon (bp)	UPIC Score	Alleles
stv-pos_03690_a	TTTAATTTTTGTGGGTAGGAGGCG	TTGCACAACATATTCGAAGCAGAG	179	-	1
stv-pos_03748_a	AAGGAGTACACCATTGCATCTTCC	CAATTAGACCTCTTGATTGCCCTG	183–195	1	3
stv-pos_03757_a	CTGCATCTTCTGCCTGCTTTTTC	ATCGCAACTTGATTTTCGACATTG	183–189	1	2
stv-pos_03767_a	AAATTTTAAGCCTTCATGCTCACC	TACCATGCCCACTTAATCCTCTTC	200–205	-	3
stv-pos_03804_a	ACTAACCTAGAGCCACTTCCCGAG	TTCCTCCTGAAGACTCAACTTTGG	168	-	1
stv-pos_03834_a	GCATCCATGGCAGATAATAGAAGG	TTTGGTACACCTATTGTTTTGCTCC	149	-	1
stv-pos_03910_a	GGCATGGATCGAAATCTTACAAAC	TTGATAACTCTGCTCCTGCACAAC	125	-	1
stv-pos_03958_a	ATAATCTGGGATTTGACATGGAGC	AAAACCACCACCACTGGTATCG	110	-	1
stv-pos_03976_a	TTCGTAATGATTTGATAGTAGTTGTTGG	AAATTTCCCTATTGCAAGGAGATG	147–151	-	2
stv-pos_03997_a	CATTCATTTGGTCAATAAGACCTCC	GCTTTGGATTCTTCACTACCCTTG	100	-	1
stv-pos_04001_a	TTTCAAGTCAAGGTTGGAAAATGC	TCAAAGCCTGGTATCACACAGAAG	121	-	1
stv-pos_04043_a	CATATGCAGTAGACAAAAGGCTTGC	TTGAACCAGTCCAAGAAGATGATG	169–182	1	3
stv-pos_04117_a	GGATCTTTGCCCAAATAACCTACC	GGGTGGAAGGGAGAAAGTTTTTAG	153	-	1
stv-pos_04126_b	TACCACGATGACCACTATCACCAC	CAAGGGAATACCAAAATGTGATGG	119–192	2	5
stv-pos_04204_a	TACCAACAATACAAGCAACAACGG	AGAACTAGGGGTGGTAAATGGGAG	177–178	-	2
stv-pos_04256_a	GTGTTTCTTTTGCCATAAAGTGGG	TTTGGTTTTCGTTTGTTTTGGAAC	129–133	1	2
stv-pos_04298_a	TCCAATTGGTGTCTACTGTTACGC	TTTGTTAGCAAGGGTTGGAATTTG	181	-	1
stv-pos_04473_a	TTTGATTGAAATATGGAGGAAAAGG	GAGGGAGGGAGATTCTCAAGG	119	-	1
stv-pos_04637_a	GACTTTTCTCCAGGCGAGGTAAC	CTAGCGGGAGAAAGTGAGAGTGAG	142–150	1	2
stv-pos_04745_a	TGCTCATGCTAATCTTGTCTTTAGTTG	TCGCATGCTTTAAGAAGATGATTG	149	-	1
stv-pos_04752_a	AAAGCACTTGCATGCGTGTATAAG	AACGTGAAGCGTTAGAAAGTGGAG	171	-	1
stv-pos_04771_a	TTTCTAGCATGGCAGTTGTAGCAC	AGCAAGGAAGGAGTACTGGAGGAG	173–188	3	6
stv-pos_04888_a	TCCTTCATCTCAAACTGGTGACTG	TATGATTCAGCCTTGTTTCTTCGC	167	-	1
stv-pos_05032_a	AATTACACACGTCCCCATTTTCTC	AAGCCATTGTAGAGAGGGAATTTG	158	-	1

Reference

1. Arias, R.S.; Ballard, L.L.; Scheffler, B.E. UPIC: Perl scripts to determine the number of SSR markers to run (Software). *Bioinformation* **2009**, *3*, 352–360.
